# Atypical Guillain-Barré Syndrome Presenting After COVID-19 Infection

**DOI:** 10.7759/cureus.29521

**Published:** 2022-09-24

**Authors:** Aleeha Noon, Jasminder K Malhi, Chung Ki Wong

**Affiliations:** 1 Internal Medicine, California Northstate University College of Medicine, Elk Grove, USA; 2 Internal Medicine, Mercy Medical Group, Sacramento, USA

**Keywords:** ascending polyneuropathy, urinary retention, intravenous immunoglobulins (ivig), covid 19, guillian barré syndrome

## Abstract

Guillain-Barré syndrome (GBS) is an acute autoimmune disease affecting the peripheral nervous system presenting as a symmetric, ascending polyneuropathy. The syndrome arises after a stimulus, such as infection or vaccination, and provokes an autoimmune response in the body. Common symptoms include rapidly progressive weakness in the extremities and generalized hyporeflexia or areflexia. However, GBS may have various presentations, which can make for a challenging diagnosis. We present a case of a 46-year-old female with asymmetric ascending weakness, paresthesias, and acute onset urinary retention occurring after Coronavirus Disease 2019 (COVID-19) infection. Of note, this patient did not present with albuminocytologic dissociation in cerebrospinal fluid (CSF) studies. The complex presentation of her symptoms prompted a diagnosis of atypical GBS. Her diagnosis was achieved through a series of diagnostic tests ruling out other etiologies, such as meningitis and spinal cord compression syndromes.

## Introduction

Guillain-Barré syndrome (GBS) is a rare autoimmune polyneuropathy that typically presents as an acute, ascending paralysis with concomitant albuminocytologic dissociation in cerebrospinal fluid (CSF) [1]. It is thought to occur due to the phenomenon of “molecular mimicry,” in which some agent, such as infection, induces an autoimmune response in the body. In the case of GBS, the agent is thought to stimulate antibodies towards Schwann cells, resulting in demyelination [2,3]. GBS is almost exclusively a peripheral neuropathy, classically manifesting as weakness of the lower extremities that quickly spreads contiguously [4]. GBS may also present with other uncommon primary symptoms, such as ophthalmoplegia, bulbar palsy, or urinary retention. Such symptoms often overlap with other diagnoses, such as strokes or spinal cord compression syndromes [5]. As a result, atypical presentations of GBS pose as a diagnostic challenge for clinicians. This case report highlights the presentation of atypical GBS following Coronavirus Disease 2019 (COVID-19) infection in a 46-year-old female patient.

## Case presentation

A 46-year-old female with known type II diabetes mellitus, hypercholesterolemia, obesity, and recent history of COVID-19 infection two months prior presented in the emergency room (ER) for evaluation of low back pain with radiation to the legs bilaterally, paresthesias in her lower legs through her buttocks, and acute onset urinary retention. The patient was seen in the clinic two weeks prior for evaluation of her lower back symptoms and numbness in her right great toe. At the time, she was diagnosed with lumbar radiculopathy. The patient also reported a history of scoliosis surgically corrected in childhood and acknowledged ongoing “on-and-off” back pain that typically subsided after a few days; however, her current flare-up never resolved. On evaluation in the clinic, she denied any urinary retention, bowel incontinence, or saddle anesthesia. She was prescribed baclofen, ibuprofen, and acetaminophen for the management of her suspected lumbar radiculopathy, which did not yield significant relief over the next two weeks.

On presentation in the ER, the patient noted worsening pain since evaluation in the clinic as well as numbness around her genitalia and rectum, which she attributed to her inability to urinate. She had been going to the bathroom multiple times with minimal to zero urine output despite the sensation of having to urinate. Straight catheterization of the bladder in the ER yielded 600 ccs of urine. She denied any history of trauma, recent injuries, or similar presentations. Vital signs in the ER remained stable. On a physical exam, she was noted to have diminished sensation to pinprick and pressure reported in the posterior aspect of bilateral lower extremities, especially over the feet and buttock regions. Additionally, she presented with a slightly diminished rectal tone with the presence of saddle anesthesia. She maintained normal strength in the bilateral lower extremities. She also displayed asymmetric deep tendon reflexes (DTRs); she had absent Achilles reflexes bilaterally and absent right knee jerk reflexes but normal upper extremity and left knee DTRs. Gait and cerebellar testing were normal. 

Her presentation was concerning for spinal cord compression, which prompted magnetic resonance imaging (MRI) and treatment with 10 mg IV dexamethasone. MRI of the cervical through lumbar spine showed diffuse disc bulge at L4-5 and L5-S1 consistent with her prior diagnosis of lumbar radiculopathy but no evidence of spinal cord compression or canal stenosis. Computed tomography (CT) myelogram was performed on the recommendation of neurosurgery and was also negative for spinal cord or nerve root compression. 

The patient was subsequently admitted for monitoring and further studies to establish a diagnosis. A summary of pertinent lab workup is provided in Table 1. Her cerebrospinal fluid (CSF) cytology was initially concerning for bacterial meningitis given elevated white blood cells with neutrophil predominance and low glucose; empiric ceftriaxone and vancomycin were thus started but soon discontinued following the result of negative cultures. CSF also revealed elevated protein, IgG oligoclonal bands, and a low-volume venereal disease research laboratory (VDRL) titer. No antibodies to myelin-basic protein or *Treponema pallidum* were present on further evaluation. She was then diagnosed with Guillain-Barré Syndrome, and treatment with intravenous immunoglobulin (IVIG) was begun to complete a five-day treatment course. Clinical improvement was noted with the patient reporting returning sensation and improving the ability to urinate. Upon completion of the IVIG course, the patient was discharged. The remainder of her treatment plan involved outpatient physical therapy and pregabalin. 

**Table 1:** Summary of pertinent laboratory results. (N): lab value is within the normal range; (H): lab value is above the normal range; (L): lab value is below the normal range; CBC: complete blood count; CSF: cerebrospinal fluid; WBC: white blood cell; Hgb: hemoglobin; RBC: red blood cell

Lab values (reference range) & Date	WBC count (4.5-11.0 × 10^9^/L)	Hgb (Female: 12.1-15.1 g/dL)	CSF glucose (50-80 mg/dL)	CSF protein (15-40 mg/dL)	Oligoclonal bands (0-1 band)	CSF RBC count (Nil)	CSF WBC count + differential (0-8/mm^3^)	CSF microbial culture (Negative)
3/6/2022	7.7 (N)	13.0 (N)	-	-	-	-	-	-
3/7/2022	9.4 (N)	12.0 (L)	-	-	-	-	-	-
3/9/2022	7.5 (N)	12.0 (L)	-	-	-	-	-	-
3/11/2022	10.1 (N)	12.7 (N)	39 (L)	177.0 (H)	-	143 (H)	165 (H), neutrophil predominance	Negative
3/12/2022	8.0 (N)	11.5 (L)	-	-	-	-	-	-
3/13/2022	7.5 (N)	11.3 (L)	-	-	4 (H)	-	-	-
3/15/2022	7.4 (N)	11.1 (L)	-	-	-	-	-	-

## Discussion

The diagnosis Guillain-Barré syndrome is typically clinical, presenting as a post-infectious ascending weakness. Another characteristic feature of GBS is its progressive, monophasic nature; in other words, symptoms do not relapse and remit [2]. A presentation outside of these general criteria is considered atypical GBS [6].

As GBS is known to occur following infection, it has been reported in cases following the novel COVID-19 infection. In contrast to the patient presented in this report, other cases of post-COVID GBS have presented akin to the classic description [7]. The patient presented in this case initially had asymmetric symptoms, with the paresthesias in her right hallux being the primary sign. Eventually, the numbness she was experiencing did progress to become equal and bilateral, similar to a classic case of GBS. However, her clinical picture was complicated by other symptoms, namely urinary retention and perianal anesthesia. These symptoms are most concerning for spinal cord compression syndromes like cauda equina or conus medullaris, both of which are neurological emergencies and require prompt imaging and treatment. Fortunately, an MRI ruled out this acute pathology. Another classic feature of GBS is a pattern of albuminocytologic dissociation on CSF studies, meaning there is an increased concentration of total protein relative to a normal total nucleated cell count [6]. GBS without this lab finding has been reported very rarely. Of note, a published case study reports normal CSF findings in a clinically-diagnosed presentation of GBS [8]. In contrast, the case we present shows elevated protein in CSF along with low glucose and an elevated white blood cell count. The latter two of these three findings created concern over possible meningitis, though this diagnosis was ruled out following a negative CSF culture. Ultimately, ruling out these more acutely morbid pathologies allowed us to arrive at the final diagnosis of an atypical presentation of GBS as the etiology for this patient’s symptoms.

Standard of care for treatment of GBS can include either IVIG or plasma exchange, both of which are supported by randomized controlled trials [9,10]. Urgent initiation of treatment is of utmost importance to prevent complications from disease progression, such as respiratory failure secondary to diaphragm paralysis from phrenic nerve demyelination [11]. Treatment with the five-day treatment course of IVIG for the patient presented in this case was successful, as evidenced by a gradual restoration of sensation, motor function, and ability to urinate independently. Prompt treatment in combination with adequate physical therapy thus proved to be an appropriate treatment course for this patient. In considering atypical variants of GBS, prompt treatment is of even greater importance since ambiguity surrounding the correct diagnosis may delay treatment and therefore permit further disease progression [12]. Hence, this case also demonstrates the importance of early recognition of both classic and atypical clinical patterns in order to appropriately diagnose and treat patients in a timely manner so as to prevent complications.

## Conclusions

In this case report, we have presented an atypical case of Guillain-Barré syndrome two months after COVID-19 infection. From the clinical presentation of this patient, we can conclude that GBS may still be an appropriate diagnosis in patients presenting with neuropathy following a stimulus such as COVID-19 infection, despite the absence of albuminocytologic dissociation in CSF studies. As demonstrated in this case, GBS may also present with non-classical symptoms, such as urinary retention and perianal numbness, thus mimicking other diagnoses like compressive cord syndromes. Hence, it is of utmost importance to rule out other serious diagnoses before assuming the presentation of GBS in a patient with ascending peripheral neuropathy.
